# Entering the golden age for antibody-drug conjugates in gynecologic cancer

**DOI:** 10.18632/oncoscience.604

**Published:** 2024-05-20

**Authors:** Michelle Greenman, Blair McNamara, Levent Mutlu, Alessandro D. Santin

**Affiliations:** ^1^Department of Obstetrics, Gynecology, and Reproductive Sciences, Yale University School of Medicine, New Haven, CT 06520, USA

**Keywords:** antibody-drug conjugates, gynecologic malignancies, precision medicine

Biologically aggressive tumors such as uterine serous carcinoma (USC) and carcinosarcoma (CS) are aggressive subtypes of endometrial cancer with a poor prognosis and a disproportionately high mortality rate [1]. Cytoreductive surgery along with chemotherapy is critical in treatment. However, recurrence is common, requiring multiple lines and combinations of chemotherapy. Use of immunotherapy in combination with gold standard chemotherapy regimens and targeted drugs represent novel modalities in treatment endowed with a remarkable potential in endometrial cancer patients. In a recent publication entitled “*In Vivo* and *In Vitro* Efficacy of Trastuzumab Deruxtecan in Uterine Serous Carcinoma” we evaluated trastuzumab-deruxtecan (T-DXd), a HER2-directed antibody drug conjugate (ADC) against biologically aggressive uterine tumors [2]. We demonstrated for the first time the remarkable preclinical activity of T-DXd against primary USC cells lines as well as USC xenografts overexpressing HER2/neu.

ADCs utilize a humanized monoclonal antibody targeting an antigen differentially expressed in tumor cells (i.e., HER2/neu for T-DXd) linked to a cytotoxic payload. The delivery of the highly cytotoxic chemotherapy agent specifically to tumor cells overexpressing the antigen is not only effective in inducing cell death but also minimizes off-target cytotoxicity. While the concept for ADCs embraced a vision for simple, effective cancer care, like with all new drugs, following the initial introduction, many difficulties were encountered. Establishing the optimal combinations of an antibody, the selection of an appropriate linker, and toxic payload proved to be a game of trial and error. Consistent with this view, several ADCs have clinically failed to demonstrate sufficient activity *in vivo* while others have encountered unanticipated side effects in human patients, forcing their use to be terminated. These challenges were reflected in only three ADCs achieving approval over 17 years after their initial introduction to patients in clinical trials.

Nevertheless, ADCs have recently become widely recognized as the future of cancer treatment, which is reflected as the fastest-growing drug class [3]. Consistent with this view, in the last few years many ADCs have been approved against a variety of solid tumors with many others moving in the pipeline. Importantly, impressive clinical outcomes with ADCs use have been seen in historically difficult to treat tumors such as USC, uterine carcinosarcomas, and platinum-resistant ovarian cancer, which have contributed to the momentum of ADC research and development. However, despite gaining popularity and establishing themselves as integral in the management of many solid tumors, ADC use in gynecologic cancers is still limited. To date, there are only two Food and Drug Administration (FDA)-approved ADCs for gynecologic malignancies (Mirvetuximab soravtansine in platinum resistant ovarian cancer and Tisotumab vedotin in recurrent or metastatic cervical cancer following progression on chemotherapy) [4, 5].

While approved ADC use in gynecologic cancers is currently limited, we anticipate this changing in the imminent future. The abundance of preclinical and clinical trials ongoing suggests we are entering into “the golden age” for ADC use in gynecologic malignancies. Accordingly, there are several ADCs under investigation with validated targets such as HER2/neu, TROP-2, CDH6, and folate receptor showing promising preclinical and clinical activity against gynecologic tumors. Our group has recently demonstrated remarkable preclinical efficacy for the ADCs trastuzumab-deruxtecan (T-DXd), DHES08151, sacituzumab govitcan, and datopotomab-deruxtecan across multiple gynecologic tumors including epithelial ovarian cancer, uterine serous carcinoma, uterine and ovarian carcinosarcoma, and cervical tumors [6–9]. Importantly, we were able to demonstrate significant bystander killing for all these ADCs, a mandatory requirement for the induction of cell death in gynecologic tumors characterized by heterogeneity in expression of the above mentioned target antigens.

Of note, only the surface has been touched on combining ADCs with other treatments, such as immunotherapies, PARP inhibitors, or traditional chemotherapy, allowing a significant space for further investigation and growth. Multimodal therapy allows for enhanced efficacy and may represent a useful strategy for overcoming resistance to chemotherapy treatment.

In conclusion, ADCs may offer targeted therapy with highly potent cytotoxic agents with reduced toxicity when compared to standard chemotherapy treatments. After decades of research, significant development has occurred and has changed the landscape of treatment in breast, non-small cell lung cancer, leukemia, and lymphomas. The introduction of Tistotumab vedotin in cervical cancer and Mirvetuximab soravtansine in platinum resistant ovarian cancer and T-DXd as agnostic indication for all unresectable or metastatic HER2-positive (IHC 3+) solid tumors who have received prior systemic treatment and have no satisfactory alternative treatment options have added to the armamentarium of available treatment, especially for advanced stage and recurrent disease. Our preclinical work paired with those of the other groups and the encouraging results of maturing clinical trials are leading way to a new wave of treatment for gynecologic cancer utilizing ADCs. We are optimistic that the incorporation of ADCs into the treatment of aggressive tumors and treatment refractory gynecologic cancers will improve quality of life and survival outcomes in our patients.
